# Colonoscopic evaluation in ulcerative colitis

**DOI:** 10.1093/gastro/gou028

**Published:** 2014-05-30

**Authors:** Elizabeth R. Paine

**Affiliations:** G.V. (Sonny) Montgomery VA Medical Center and Division of Digestive Diseases, Department of Medicine, University of Mississippi Medical Center, Jackson, MS, USA

**Keywords:** ulcerative colitis, colonoscopic evaluation, disease activity, mucosal healing

## Abstract

Colonoscopic evaluation is an important tool in the evaluation of ulcerative colitis (UC). UC is divided by disease extent into proctitis, proctosigmoiditis, left-sided colitis, and pan-colitis. In addition, a cecal or peri-appendiceal patch and backwash ileitis are associated with UC. The extent and behavior of UC has been characterized further using various indices and scoring systems; among these systems is the Mayo Score, which is widely used in current clinical trials for new medications. As these medical therapies for UC have developed, achieving mucosal healing with medications has become an important therapeutic objective.

## INTRODUCTION

Ulcerative colitis (UC) is a form of inflammatory bowel disease in which endoscopy plays a vital role in diagnosis, differential diagnosis, disease monitoring, and dysplasia surveillance.

## DIAGNOSIS AND ASSESSMENT OF DISEASE EXTENT

Among the common endoscopic features of UC seen within the colon are edema, erythema, mucosal friability and bleeding, erosions and ulcerations, and loss of the typical vascular pattern [1]. These features are seen within an anatomical disease extent, categorized endoscopically into proctitis, proctosigmoiditis, left-sided colitis, and extensive colitis [2]. Other endoscopic designations associated with UC include backwash ileitis and a cecal or peri-appendiceal patch [3]. Distinctions according to the proximal extent of inflammation are important because of their implications for colon cancer risk and other complications [4]. In addition, the distribution of inflammation can change over time, with the usual extension progressing from proctitis along a continuum to extensive colitis; in one review, up to 28% of patients at 10-year follow-up had extension of inflammation from their initial disease location [5].

### Proctitis

**Figure 1.**
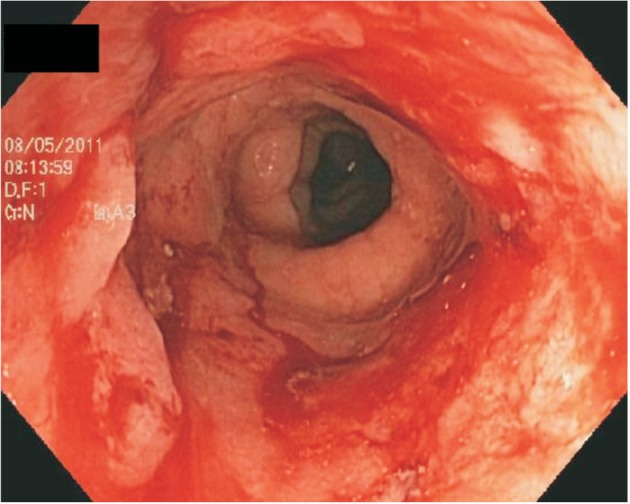
Ulcerative proctitis.

Ulcerative proctitis is the term given to inflammation confined to the rectum [6] (Figure 1). By the Montreal Classification, ulcerative proctitis is designated as E1 [7]. Patients with ulcerative proctitis commonly present with rectal bleeding or occult blood in their stools [8]. Due to these symptoms, patients undergo a flexible sigmoidoscopy or colonoscopy with biopsy which then reveals inflammation limited to the rectum on endoscopy or histology. The first-line therapy for ulcerative proctitis is a topical suppository, which allows the drug to act directly on the inflamed mucosa of the rectum [9]. Specifically, by the European Crohn’s and Colitis Foundation (ECCO) guidelines, the initial management of mild-to-moderate proctitis is 1 gram mesalazine suppositories once daily, with mesalazine foam enemas as another option, albeit less effective. In cases in which additional treatment is needed, combined management with topical mesalazine and oral mesalazine or topical steroids is recommended [10].

### Proctosigmoiditis

Proctosigmoiditis denotes inflammation in the rectum and rectosigmoid colon [6]. An estimated 25–75% of newly-diagnosed cases of UC are confined to the rectum and rectosigmoid colon [11, 12]. Rectal enemas of mesalamine or hydrocortisone are used for the treatment of this distribution of inflammation [6].

### Left-sided colitis

Left-sided colitis is the term used for inflammation extending from the rectum proximally to the splenic flexure [4]. This disease extent is known as E2 by the Montreal Classification [7]. Approximately 20–30% of patients with UC have inflammation in this disease distribution. According to the ECCO guidelines, mild-to-moderate left-sided colitis should initially be managed with 1 gram 5-aminosalicylate (5-ASA) enemas daily, in addition to more than 2 grams of oral mesalazine daily [10]. If escalation of therapy is needed due to continued or worsening symptoms, systemic corticosteroids are recommended, with hospital admission for severe disease [10].

### Extensive colitis

Extensive colitis denotes extension of inflammation proximal to the splenic flexure [13]. In the Montreal Classification, extensive colitis is known as E3 [7]. This term is also used for colitis that extends throughout the entire colon [4]. Mild-to-moderate extensive colitis is initially treated with 5-ASAs, while severe or refractory extensive colitis is treated with oral or intravenous corticosteroids or anti-tumor necrosis (anti-TNF) agents [14]. In mild-to-moderate disease, at least 2 grams of oral 5-ASAs and topical mesalazine should be used [10]. Eventually, up to a third of patients with extensive colitis will need a colectomy for definitive treatment [4].

### Backwash ileitis

**Figure 2.**
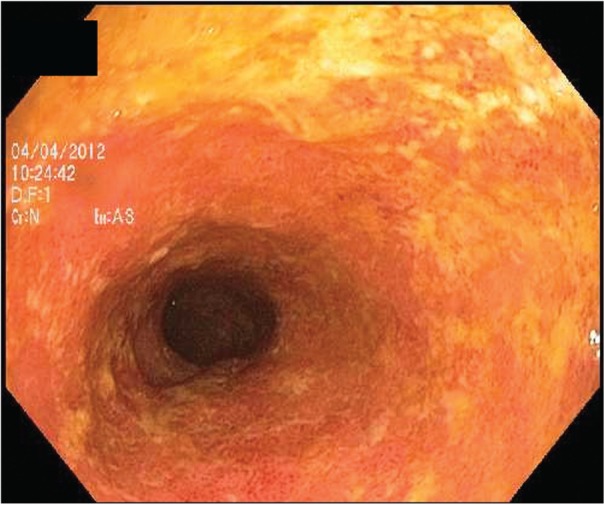
Backwash ileitis in a UC patient.

**Figure 3.**
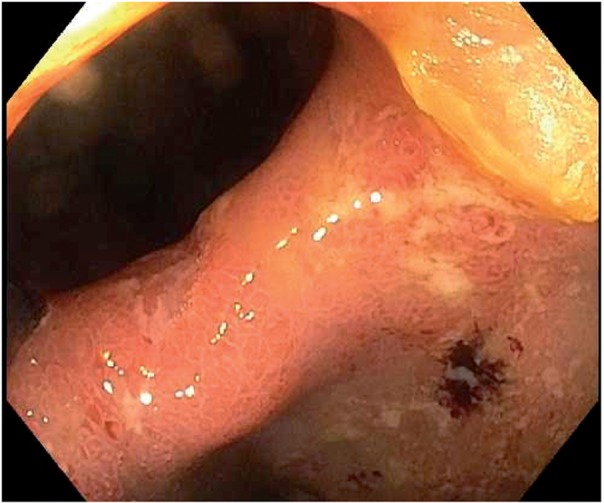
Backwash ileitis with a widely patent ileocecal valve in a UC patient.

Backwash ileitis is the term given to endoscopic and/or histological inflammation that extends from the cecum continuously into the terminal ileum in a UC patient with extensive colitis [15]. The pattern of mucosal inflammation of the right colon and the terminal ileum is often similar [16], with a widely patent ileocecal valve. Backwash ileitis can be distinguished endoscopically from the ileitis seen in Crohn’s disease (CD) by the absence of strictures or distinct ulcers in the ileocecal valve and/or terminal ileum (Figures 2 and 3)—both features more commonly associated with CD ileitis [3]. Backwash ileitis is common in UC patients with concurrent primary sclerosing cholangitis (PSC) [15]. Backwash ileitis has been reported in 10–20% of colectomy specimens of patients with extensive colitis [17, 18]. Patients with this type of inflammatory pattern of UC tend to be younger and tend to have disease that may be more difficult to treat, with surgery often required earlier in their clinical courses [19].

### Cecal or peri-appendiceal patch

A cecal or peri-appendiceal patch denotes an area of inflammation in the cecum surrounding the appendiceal orifice, which is generally discontinuous from the remainder of colonic inflammation in UC; these patients have normal right-sided colonic mucosa without inflammation [20]. Although this lesion is considered a ‘skip lesion', it is regarded as a subset of UC, rather than as a type of CD [21]. The presence of this phenomenon has been reported in 15–75% of UC patients during endoscopy or during surgical pathology evaluation of colectomy specimens in various studies [22–24].

It should be pointed out that disease distribution in UC can become patchy, even with an endoscopic appearance of ‘rectal sparing' after topical, oral, or intravenous medical therapy [3]. The patient can mistakenly be labeled as having CD; therefore it is important to document endoscopic and histological features of the terminal ileum, colon, and rectum during the very first colonoscopy (i.e. the index colonoscopy) before initiation of medical therapy [3].

## ASSESSMENT OF DISEASE ACTIVITY

Disease activity in UC has been extensively evaluated using various tools incorporating both clinical and endoscopic features. These scoring systems have been developed in an attempt to evaluate systematically the responses to treatments being studied in UC patients [25]. The most commonly used scoring system for endoscopic disease activity in recent trials is the Mayo Score. Other indices that have less commonly been used include the Ulcerative Colitis Endoscopic Index of Severity (UCEIS) score, Baron Score, Ulcerative Colitis Colonoscopic Index of Severity (UCCIS), Rachmilewitz Endoscopic Index, Sutherland Index, Matts Score, and Blackstone Index.

### The Mayo Score

The Mayo Score is a combined endoscopic and clinical scale used to assess the severity of UC. This score was first proposed by Schroeder *et al.* [26] in 1987 in a clinical trial of 5-ASA drugs in UC and has been used in various subsequent clinical trials and clinical practices. The Mayo Score is a composite of subscores from four categories, including stool frequency, rectal bleeding, findings of flexible proctosigmoidoscopy or colonoscopy, and physician’s global assessment, with a total score ranging from 0–12 [26]. Within the endoscopic component of the Mayo Score, a score of 0 is given for normal mucosa or inactive UC, while a score of 1 is given for mild disease with evidence of mild friability, reduced vascular pattern, and mucosal erythema. A score of 2 is indicative of moderate disease with friability, erosions, complete loss of vascular pattern, and significant erythema, and a score of 3 indicates ulceration and spontaneous bleeding [26]. Mucosal healing has been defined as a Mayo endoscopic subscore of 0 or 1 in major trials of biological therapies in UC including ACT-1 and ACT-2 [27], ULTRA-1 [28], ULTRA-2 [29], PURSUIT-SC [30], and PURSUIT-M [31].

### The Ulcerative Colitis Endoscopic Index of Severity

The Ulcerative Colitis Endoscopic Index of Severity (UCEIS) is a newer endoscopic scoring system that includes an assessment of vascular pattern, bleeding, and ulcers and excludes mucosal friability. In this system, the vascular pattern is rated as 1–3 with 1 as normal, 2 as patchy loss of vascular pattern, and 3 as complete loss of vascular pattern [32]. Bleeding is characterized from 1–4 with 1 as none, mucosal bleeding as 2, mild colonic luminal bleeding as 3, and moderate or severe luminal bleeding as 4 [32]. Erosions and ulcers are characterized from 1–4 with 1 as none, 2 as erosions, 3 as superficial ulcerations, and 4 as deep ulcers [32].

### The Baron Score

Among other endoscopic scoring systems are the Baron Score and Modified Baron Score. In the Baron system, patients are given a score between 0 and 3 with 0 representing normal mucosa with no bleeding and normal vascular pattern present throughout the colon; in addition, a score of 1 represents abnormal mucosa that is not expressly hemorrhagic [33]. A score of 2 is given for bleeding with light intervention with an instrument of the mucosa but no spontaneous bleeding, while 3 is given to spontaneous bleeding before the instrument is introduced [33]. Endoscopic remission is defined as a Baron Score of ≤1 [33].

The Modified Baron Score consists of a scale of 0–4 with 0 representing normal mucosa without friability and 1 representing hyperemia and granular mucosa with loss of vascular pattern and without friability [34]. A score of 2 is similar to 1, except that the mucosa is friable without spontaneous bleeding, while a score of 3 is similar to 2 with the addition of spontaneous mucosal bleeding. A score of 4 is similar to 3 with the addition of ulceration and denuded mucosa [34]. A score of 0 points is designated as endoscopic remission [34].

### The Ulcerative Colitis Colonoscopic Index of Severity

The Ulcerative Colitis Colonoscopic Index of Severity (UCCIS) is another endoscopic scoring system. In a study by Thia *et al.* [35], vascular pattern, ulcerations, bleeding-friability, and granularity were all found to have good-to-excellent intra-observer agreement in predicting overall endoscopic severity; these components were used to make the UCCIS. Patients with a normal vascular pattern were given a score of 0, while those with a partial loss of pattern were given a 1, and patients with complete obliteration of vascular pattern were given a 2 [35]. Ulcerations were graded as absent (0), erosions or pinpoint ulcers (1), multiple shallow ulcers with mucus (2), deep ulcers (3), or diffuse ulcers involving more than 30% of the mucosa (4) [35]. In terms of bleeding and friability, mucosa with no bleeding or friability was designated 0, while mucosa with friability and bleeding with minimal touch was rated 1, and tissue with spontaneous bleeding was given 2 [3]. Granularity was divided into 0–3, with 0 corresponding with no granularity, 1 with fine granularity, and 2 with coarse granularity [35].

### The Rachmilewitz Endoscopic Index

The Rachmilewitz Endoscopic Index uses scores ranging from 0–3 based on granulation, vascular pattern, mucosal vulnerability, and mucosal damage [36]. If granulation tissue is not present, a score of 0 is given, while its presence results in a score of 2 [36]. Vascular pattern is characterized as normal (0), faded (1), or absent (2), while vulnerability of mucosa is scored as having no bleeding (0), having contact bleeding (2), and having spontaneous bleeding (3) [36]. Mucosal damage—such as erosions and ulcers, mucus, and fibrin—is characterized as none (0), mild (2) or significant (3) [36]. Endoscopic remission is defined as ≤4 points by this index [36].

### The Sutherland Index

The Sutherland Index—also known as the UC Disease Activity Index or UCDAI—is a combined clinical and endoscopic scoring system used with UC patients. The endoscopic portion is scored from 0–3 and evaluates for friability, exudates, and spontaneous hemorrhage [37]. A score of 0 is given for normal mucosa, while scores of 1 and 2 represent mild and moderate mucosal friability, respectively [37]. A score of 3 represents spontaneous hemorrhage [37]. Endoscopic remission is defined as a score of 0 by this index [37].

### The Matts Score

The Matts Score is based on the granularity, bleeding, and ulceration of the colonic mucosa. A score of 1 is given for normal mucosa, while a score of 2 is given for mild mucosal granulation with mild bleeding with intervention with an instrument [38]. A score of 3 is given for significant mucosal granularity and edema with contact and spontaneous bleeding, and a score of 4 is given for severe mucosal ulceration and hemorrhage [38].

### The Blackstone Index

The Blackstone Index is divided into four categories, each further divided into two subcategories [39]. Quiescent disease is characterized by an abnormal or obliterated vascular pattern (1) or by granularity (2). Mildly active disease is characterized by focal or continuous erythema (3) or contact-induced bleeding (4) [39]. Moderately active disease is characterized by the presence of mucopurulent exudate (5) or less than 10 ulcers (<5 mm in size) per 10 cm section (6) [39]. Severe colitis is characterized by ulcers >5 mm in size with more than 10 per segment (7) or spontaneous bleeding (8) [39].

### Comparisons of scoring systems

Although many of these scoring systems have been utilized in clinical trials and gastroenterology practices, most of them are not validated. The Mayo Score, Modified Baron Score, Rachmilewitz Endoscopic Index, Sutherland Index, Matts Score, and Blackstone Index have not been validated [25]. On the other hand, the UCCIS has been validated and was found to be reproducible in a study by Samuel *et al.* [40], which found good-to-excellent inter-observer agreement in the four mucosal abnormality components of the score [40]. The UCEIS has also undergone initial validation, with findings of good intra-investigator agreement (Kappa = 0.72) and moderate inter-investigator agreement (Kappa = 0.50) in UCEIS score designation; however, further validation of this score has been recommended [41].

These assessments of disease activity have various strengths and weaknesses: the simplicity of calculation is a major strength of the Baron Score, Mayo Endoscopic Subscore, Modified Baron Score, and Rachmilewitz Index [42]. Another strength of the Mayo Endoscopic Subscore and Modified Baron Score is that these scores are used in clinical trials [42]. Strengths of the UCEIS and UCCIS include the rigorous methods used to develop them, as well as their accuracy [42]. A weakness of the Baron Score and Sutherland Index is the exclusion of evaluation for ulcers; in addition, the Modified Baron Score does not distinguish between superficial and deep ulcers [42]. Additional weaknesses in these scores are the inclusion of subjective assessments of bleeding in the Rachmilewitz Index and Baron Score and the lack of accurate distinction of friability between mild and moderate in the Sutherland Index and Mayo Endoscopic Subscore [42].

## ENDOSCOPIC MUCOSAL HEALING

Mucosal healing has become an important concept in the management of IBD patients. Achieving this endpoint in IBD therapy is based on complicated physiological processes involved in the reduction of intestinal inflammation and in the bolstering of intestinal barriers [43]. Anti-TNF agents—such as infliximab—have been shown to result in ultrastructural changes involved in mucosal healing in as few as 4 weeks [44]. The clinical importance of these ultrastructural changes, as well as the histologic and endoscopic changes involved in mucosal healing, is still evolving as new research evaluates each of these areas.

### Definition and current concept of mucosal healing

The definitions of mucosal healing have varied throughout recent medical literature, and there is currently no validated consensus on the matter. Mucosal healing has traditionally denoted the absence of visible ulcers on endoscopy, a definition that is more applicable to CD than UC, since the mucosa in UC often lacks ulcers [43]. Another definition of mucosal healing, proposed by D’Haens *et al.* [25], is endoscopic remission without blood, ulcers, erosions, or friability in each segment examined on endoscopy. Other definitions include improved endoscopic features, particularly in previously inflamed areas; normal mucosa with pseudopolyps; and histological healing [45]. In trials of therapeutic agents, mucosal healing has been defined as a Mayo Endoscopic Subscore of 0 or 1 after therapy, in patients who scored 2 or more before [31, 46].

As the definition of mucosal healing is in flux, so are the current concepts related to mucosal healing, underscoring the need for a standardized definition for use throughout the clinical and scientific communities. One issue that has arisen with the use of endoscopic remission as the definition of mucosal healing is the need to assume that the same mucosa that is normal on a current endoscopy was formerly inflamed; this definition requires that previous endoscopies be carefully compared with the latest findings [47]. Another issue is that if mucosal healing is defined as histologic remission, an assumption must be made that the pathologist’s interpretation of inflammation is objective [47]; however, in one study, only 75% of patients with UC were identified by experienced pathologists using established criteria [48].

### Clinical aspects of mucosal healing

Mucosal healing has been shown to differ from symptomatic or clinical resolution of disease activity. Various studies have shown that even when the patient has no symptoms, endoscopic and histologic evidence of disease activity in the rectum can still be present [49, 50], indicating a lack of mucosal or histologic healing. Other studies have shown that patients who have acute inflammation on rectal biopsies are more likely to relapse in the next year, even if they are in clinical remission [51].

On the other hand, mucosal healing has been associated with a reduced rate of colectomy. In one study including 448 UC patients with at least one year of follow-up, mucosal healing was associated with significantly reduced rates of colectomy [52]. In another analysis of the ACT-1 and ACT-2 trials, patients with mucosal healing while taking infliximab, with a Mayo Endoscopic Subscore of 0 or 1 at Week 8 had a reduced risk of colectomy in the subsequent year in comparison with patients with scores of 3 or 4; in addition, patients with a Mayo Endoscopic Subscore of 0 at Week 8 had a significantly higher rate of steroid-free remission at Week 54 and had higher rates of symptomatic relief at Weeks 30 and 54 than those with scores of 1 at Week 8 [46]. Such results suggest that the future activity of UC can be predicted by mucosal healing [43].

Mucosal healing has also been associated with other improved outcomes; for example, mucosal healing has been associated with a lower rate of relapse of disease. In a study by Wright *et al.* at 1-year follow-up, 40% of patients with mucosal healing while taking corticosteroid therapy did not relapse, as opposed to 18% of patients without mucosal healing [53]. In another study, among patients in clinical remission after receiving six weeks of oral and rectal mesalazine, significantly fewer patients who had mucosal healing at their subsequent colonoscopy experienced clinical relapse at 1 year, compared with patients without mucosal healing at their subsequent colonoscopy (23% vs 80%; *P* < 0.0001) [54]. Mucosal healing is also thought to be a predictor of decreased risk for colon cancer in UC patients; in a case-control study, authors found that patients with UC and colon cancer had significantly lower rates of previous or current mucosal healing during the study period (odds ratio 0.40; 95% confidence interval 0.21–074); in addition, in this study, UC patients with endoscopically normal mucosa had a similar 5-year rate of colon cancer as the general population [55].

### Mucosal healing as a therapeutic target

**Figure 4.**
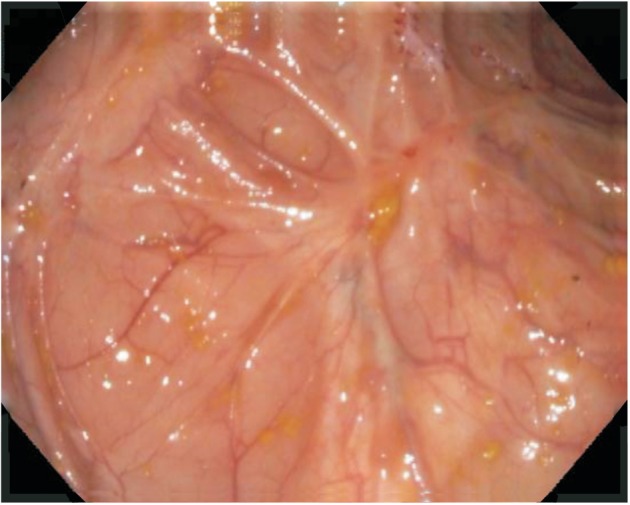
Mucosal healing after effective therapy.

Several non-biological medications used in the treatment of UC have been shown to result in mucosal healing (Figure 4); mesalazine is one of these. In pooled analysis from the ASCEND I and ASCEND II trials of delayed release oral mesalazine, 80% of patients with moderately active UC, given 4.8 grams/day of mesalazine for six weeks, had documented mucosal healing, compared with 68% of those given 2.4 grams/day of mesalazine for the same time period (*P* = 0.012) [56]. In a Cochrane review of 5-ASA use in ulcerative colitis, pooled analysis of four trials showed that patients receiving placebo were more likely to fail to achieve endoscopic remission than patients receiving 5-ASA therapy (66% vs 50%, respectively) [57]. In another study, 7 of 21 patients with chronic relapsing UC experienced mucosal healing with sulfasalazine, and nine experienced partial colonoscopic remission with this therapy; in the same study, 11 of 21 patients with chronic relapsing UC who received olsalazine had mucosal healing, with nine patients experiencing partial endoscopic remission [58].

Systemic corticosteroids have also been evaluated in regard to mucosal healing. One study showed that within 6 weeks, oral steroids at 100 mg daily resulted in a 30% rate of mucosal healing, in comparison to 10% for placebo [59]. In another study, patients who did not achieve mucosal healing on corticosteroid therapy had a 48.7% rate of combined negative endpoints (hospitalization, immunosuppression therapy, and colectomy), compared with 26.7% in those who experienced both mucosal healing and clinical remission on corticosteroids. In this same study, multivariate analysis of factors resulting in negative outcomes at 5 years showed that the only factor was the lack of mucosal healing [60].

The use of thiopurines also leads to mucosal healing. In a study by Ardizzone *et al.*, significantly greater numbers of patients receiving azathioprine (AZA) for 6 months experienced mucosal healing and clinical remission in comparison to those receiving 5-ASA medications for the same time period (53% vs. 19%, p=0.006). In another study, colonoscopies were performed on 20 UC patients who received thiopurine therapy for at least one year and who exhibited clinical remission on this treatment; of these patients, 60% achieved mucosal healing, as defined by a Mayo Endoscopic Subscore of 0, and 22.2% of patients experienced relapse in the follow-up period [61]. Furthermore, in a study by Paoluzi *et al.*, of 32 patients on AZA 2 mg/kg/day, 24 patients showed endoscopic remission at 6 months, with 22 of these patients achieving histologic remission during this time period [62].

Biological therapies have also been shown to induce mucosal healing. In the ACT-1 study, 62% of patients receiving infliximab at 5 mg/kg showed mucosal healing at Week 8, as opposed to 34% of patients receiving placebo. Similarly, in the ACT-2 trial, 60% of patients receiving 5 mg/kg infliximab had mucosal healing at Week 8, in comparison to 31% of those receiving placebo [27]. Furthermore, in the ULTRA-1 trial, the rate of mucosal healing was 46.9% in the group receiving adalimumab 160 mg at Week 0 followed by 80 mg at Week 2 and 40 mg at Weeks 4 and 6. This contrasts with the 41.5% rate of mucosal healing in the placebo group [28]. In the ULTRA-2 trial, adalimumab was shown to induce mucosal healing at 8 weeks in 41% of patients receiving adalimumab, compared with 32% of those receiving placebo; in addition, the combined rate of mucosal healing in patients on adalimumab at Weeks 8 and 52 was significantly higher than for placebo (18.5% vs 10.6%; *P* = 0.013) [29]. Finally, the new subcutaneous anti-TNF agent golimumab has been shown to result in mucosal healing. In the PURSUIT-SC trial of patients receiving 400 mg golimumab at Week 0 and 200 mg at Week 2, 45% had mucosal healing, compared with 29% of those receiving placebo. In addition, of patients receiving 200 mg of golimumab at Week 0 and 100 mg at week 2, 42% had mucosal healing, in contrast to 29% who received placebo [30].

## CONCLUSION

Ulcerative colitis is a complex disease characterized endoscopically based on its mucosal features, disease extent, and disease activity. The mucosal changes are seen within a particular anatomical distribution of the colon or distal small bowel. Colonoscopic evaluation of the mucosa is crucial so that disease activity can be assessed according to various indices and so that therapy targeted for particular segments of the colon can be prescribed. Colonoscopy also allows for evaluation of endoscopic remission, which has been associated with mucosal healing. Mucosal healing is an important emerging concept in IBD management, but there is currently no standardized definition of the term, and various studies use different definitions. Despite this heterogeneity in definition, it is clear that mucosal healing results in improved outcomes and most of our currently available UC therapies result in mucosal healing—at least to some degree. In the future, a standardized definition of mucosal healing will probably be developed, and this concept will continue to evolve. Colonoscopy will continue to be an important part of this process as our understanding of UC and its management improves.

**Conflict of interest:** none declared.
